# Using an Adult-Designed Wearable for Pediatric Monitoring: Practical Tutorial and Application in School-Aged Children With Obesity

**DOI:** 10.2196/76166

**Published:** 2026-03-20

**Authors:** Alessandra Angelucci, Andrea Aliverti, Matteo Vandoni, Daniela Lucini, Cristiana Larizza, Vittoria Carnevale Pellino, Gianvincenzo Zuccotti, Valeria Calcaterra

**Affiliations:** 1 Dipartimento di Elettronica, Informazione e Bioingegneria, Politecnico di Milano, Piazza Leonardo da Vinci 32, Milan, 20133, Italy, +39 3204299516; 2 Laboratory of Adapted Motor Activity (LAMA), Department of Public Health, Experimental Medicine and Forensic Science, University of Pavia, Pavia, Italy; 3 BIOMETRA Department, University of Milan, Milan, Italy; 4 Exercise Medicine Unit, Istituto di Ricovero e Cura a Carattere Scientifico Istituto Auxologico Italiano, Milan, Italy; 5 Department of Computer, Electrical and Biomedical Engineering, University of Pavia, Pavia, Italy; 6 Department of Biomedical and Clinical Science, University of Milan, Milan, Italy; 7 Pediatric Department, Buzzi Children's Hospital, Milan, Italy; 8 Department of Internal Medicine and Therapeutics, University of Pavia, Pavia, Italy

**Keywords:** childhood obesity, wearable devices, activity monitoring, cardiovascular monitoring, wearable data quality

## Abstract

This tutorial presents a step-by-step guide on how to use an adult-oriented wearable (Fitbit) to collect and analyze activity and cardiovascular data in a pediatric population of school-aged children with obesity. After outlining the current landscape of commercial wearables for adults and children, the tutorial illustrates the distinct considerations required for accurate pediatric monitoring, especially for cardiovascular metrics and derived features. The text provides a clinical application, highlighting how data from these devices were gathered and integrated with standard clinical measurements (ie, 1 week of monitoring with the wearable compared with the 6-minute walk test). The tutorial also discusses potential correlations, which should be interpreted as exploratory, given the small sample size (n=16), as well as limitations and future perspectives on using wearables for long-term pediatric monitoring of school-aged children, aiming to inform clinicians, researchers, and other stakeholders about the additional considerations that are needed to use wearables designed for adults to monitor this age group.

## Introduction

### Wearable Devices

Wearables are considered key elements of future health care because they enable ecological measurements without interfering with daily life. Feedback provided by consumer devices, especially those equipped with displays, can induce behavioral reactivity, potentially nudging toward active lifestyles [1]. Wearables find many applications in the field of telemonitoring of patients [2], consumer health and well-being, sports performance assessment (particularly in endurance disciplines and predominantly during training rather than competition [3]), environmental monitoring [4], and emergency settings [5]. Examples of wearables include watches (such as smartwatches and activity trackers), eyeglasses [6], visors [7], rings [8], patches [9,10], sensorized garments (such as shirts, gloves, and socks) [11], elastic bands applied to the torso [12,13], earbuds [14], and devices clipped to the shoes [15].

Even if it is often unclear to what extent nonmedical-grade commercial devices are validated, such devices have been proposed in the medical field as self-assessment tools [16,17] and may provide data associated with information of interest, such as activity levels and physiological parameters.

Activity metrics range from the number of steps performed in a fixed period [18], most commonly in a day, to a wide variety of parameters, such as distance, minutes in different heart rate zones (HRZ), and the number of stand hours [19].

Physiological measurements can consider a single parameter, for example, heart rate (HR) or pulse rate (PR) [20], peripheral blood oxygen saturation [20], respiratory rate [21], skin temperature, and galvanic skin response or electrodermal activity, or could be more complex measures combining data from several sensors, such as in the case of sleep analysis, estimation of energy expenditure, and analysis of physiological parameters with respect to activity [22-25].

### Devices for School-Aged Children

A first distinction must be made between wearable systems for neonates, infants, and school-aged children. Certain US Food and Drug Administration–cleared medical-grade patches, such as ANNE One (Sibel Health Inc) [26,27], have been successfully deployed for continuous vital sign monitoring in neonates and infants, particularly in intensive care settings [27]. These devices are designed for short-term clinical use under medical supervision and are optimized for the anatomical and physiological characteristics of newborns (eg, very small body size, high resting HR, thin skin, and limited mobility). However, such solutions are not directly applicable to ambulatory or home-based pediatric monitoring in school-aged children. Children exhibit larger body dimensions, mobility, and behavioral variability, which require wearables that are robust, reusable, and comfortable for daily use outside the hospital environment.

Another distinction should be made between devices for adults and children. Physiological monitoring in the school-aged child population is inherently more complex than in adults due to several factors. First, children have markedly different cardiovascular physiology; for instance, normal HRs in healthy children can reach upward of 180 beats per minute [28]. Second, anatomical scaling poses challenges in terms of device design, as wearables must be smaller and adapt to smaller vessels, thinner tissues, variable subcutaneous fat, and smaller chest circumference. Third, children’s patterns of movement and physical activity are more dynamic and unpredictable, which increases motion artifacts, signal noise, and intermittent sensor loss [29]. Failure to monitor cardiovascular metrics continuously in children can delay early detection of hemodynamic compromise, mask subtle progression of dysfunction, and limit timely interventions, with consequences for morbidity and long-term outcomes [30]. The limitations of wearables for pediatric monitoring add up to the already existing limitations of wearable devices, even more so when commercial devices are involved.

In general, the results of studies about the accuracy and precision of commercial smartwatches used by adults are inconsistent. Regarding PR measurement from a Fitbit wrist-worn device, a study [31] found that the accuracy of PR estimation from a photoplethysmographic (PPG) sensor (<10% mean absolute error with respect to a gold-standard electrocardiogram) across 24 hours and during various activities was acceptable. Conversely, other studies [32] reported significant accuracy variations depending on the activity type, and a notable proportion of PR measurements were substantially inaccurate. Focusing on physical activity metrics, a 2015 study [33] found that Fitbit’s step counts strongly correlated with those obtained by gold-standard methods. However, they observed low accuracy at slower walking speeds. Additionally, such results are valid in the adult population and cannot be immediately translated to pediatric applications.

It becomes even more complicated when children are involved. In fact, most research on the use of wearables in children is focused on activity tracking [34]. At the market level, popular models of wearables for children are limited in the number of integrated sensors and, therefore, in functionality. Most models only perform the function of activity trackers, such as the Fitbit Ace 3 [35] and the Garmin vívofit jr. 3 [36]. The Fitbit Ace 3, for instance, is only equipped with an accelerometer and not with a PPG sensor, thus making it not usable to assess cardiovascular features. Brands such as Apple and Polar do not offer any consumer product that is specifically designed for children. Polar used to offer the Polar Active [37] device with a business-to-business model for physical activity programs in school-aged children [38].

Nash et al [39] evaluated the accuracy of Apple Watch–derived cardiac features in children, both for the PPG and electrocardiography (ECG). In terms of PPG, they assessed whether PR estimates were accurate during both sinus rhythm and arrhythmias and found that there was a good agreement between the PPG and the gold-standard ECG during sinus rhythm (intraclass correlation coefficient [ICC] 0.98‐0.99) and poor agreement during arrhythmias (ICC 0.24‐0.27).

In a prospective cohort of children referred for 24-hour Holter monitoring (n=31, mean age 13.2, SD 3.6 y), a PPG-based device with the European Union Medical Device Regulation approval for use in adults, CardioWatch 287-2B (Corsano Health BV), achieved a mean accuracy of 84.8% (SD 8.7%) of readings; accuracy was significantly higher at lower HR (mean 90.9%, SD 9.3%) than at higher HR (mean 79.0%, SD 10.6%), and Bland–Altman analysis showed a bias of −1.4 bpm with 95% limits of agreement from −18.8 to +16.0 bpm [40].

In addition, ECG has been explored in smartwatches for pediatric applications. Unlike optical PPG-based wearables, the ScanWatch (Withings) uses a one-lead ECG approach. In a study of 100 children aged 5 to 17 years, the ICC for HR was 0.97, 0.86 for the PR interval, and 0.80 for the QT interval, whereas QRS and QTc interval agreement was only moderate [41].

### Rationale and Aim of This Tutorial

The market for wearables for pediatric monitoring of school-aged children presents a gap in the offer and availability of monitors of cardiovascular function, as the most popular commercial wearable brands do not offer PPG-equipped (or ECG-equipped) devices for children or do not offer devices for children at all. It is nowadays necessary to use adult-designed wearables if there is a need for cardiovascular monitoring. The rationale for this tutorial focuses on the methodological adaptation required to use adult-grade devices equipped with PPG sensors and other integrated modules for cardiovascular monitoring in school-aged children. Dedicated child-specific devices with comparable sensing capabilities are largely unavailable, and even when they are marketed as child-specific devices, it is often not disclosed whether the changes are only aesthetic and in shape or also from a sensors and algorithms point of view. This tutorial, therefore, highlights both the opportunities and the technical and physiological limitations of repurposing such devices for pediatric research and clinical applications. Rather than testing clinical hypotheses, the primary aim of this work is to demonstrate methodological feasibility by showing how an adult-designed wearable can be deployed, processed, and interpreted when used in school-aged children and by providing a transparent, reproducible workflow for researchers and clinicians. The clinical example included in the manuscript serves to illustrate the application of these methods, not to evaluate the effectiveness of the wearable or to draw population-level clinical inferences.

## Step-by-Step Tutorial

### Overview

The workflow is organized into 12 steps that readers can replicate in pediatric studies using adult-designed wearables. Each step describes what to do and how to do it, including defining the clinical context (steps 1‐4), managing pediatric-specific reference values (step 5), selecting and importing Fitbit files (steps 6 and 7), extracting activity and cardiovascular metrics (steps 8‐10), assessing data quality (step 11), and conducting statistical analysis (step 12).

### Step 1: Framing the Clinical Case

The first step must be taken before collecting any data and consists of framing the clinical case that will be investigated by using adult-designed wearables in a pediatric population. This is a necessary step that guides the future choices of the parameters that will be retained or discarded and the clinical outcomes.

This tutorial includes a practical example based on data collected from children with obesity to demonstrate how wearable-derived metrics can be dealt with and compared with conventional clinical measures when adult-grade devices are used in children. Childhood obesity, defined according to the World Health Organization growth reference as a BMI greater than +2 SDs above the age- and sex-specific mean [42], represents a major global health concern [43]. BMI remains the most widely used indicator for classification, with equivalent percentile-based criteria also adopted by the Centers for Disease Control and Prevention [44]. By including this clinical case, the tutorial aims to illustrate how data extracted from an adult-designed wearable can be contextualized and analyzed in relation to standard clinical outcomes, such as the 6-minute walk test (6MWT) and blood pressure percentiles.

Exercise, as a nonpharmacological approach, can help delay obesity-related conditions, improve cardiovascular health, and regulate inflammation in both children and adults [45]. In addition, exercise promotes improvement in functional capacity, which is also reduced in the pediatric population with obesity [46].

Traditional tests and measures of functional capacity evaluate different aspects of physical activity and exercise tolerance; some are subjective (eg, evaluation scales), while others provide objective or quantitative data. Among the objective tests, the 6MWT [47] is considered a simple field-based approximation of submaximal exercise capacity, whereas cardiopulmonary exercise testing represents the gold standard for assessing maximal aerobic capacity but is expensive and time-consuming and thus reserved for selected cases [48,49]. In particular, the 6MWT evaluates exercise tolerance and consists of making the patient walk at a self-paced speed up and down a 30-m-long corridor for a fixed period of 6 minutes [47], and the total distance traveled in meters (6MWT distance or 6-minute walk test distance) is the primary result of this test.

Intermediate approaches such as exercise electrocardiogram stress testing [50] offer additional physiological insight but require clinical supervision. It is also worth noting that in nonclinical or educational contexts, such as school-based physical activity programs, functional capacity is often assessed using simpler field tests (eg, shuttle run or step tests), which provide practical but less accurate estimates of exercise tolerance than laboratory-based evaluations (eg, 6MWT and cardiopulmonary exercise testing) [51].

### Step 2: Ethics Committee Approval

The second step consists of obtaining approval to collect data with adult-designed wearables in a cohort of children chosen based on the specific clinical case, which has already been defined in step 1. Approval from an ethics committee or institutional review board is required, and compliance with local laws on the privacy of minors is up to the investigators. To obtain this approval, it is necessary to outline the cohort and the experimental procedures to be followed. The same dataset is generally used for more than one research aim.

In this case, data were obtained from a cohort of children with obesity studied before and after a 12-week online exercise training program, the clinical outcomes of which were published by Calcaterra et al [52]. The study was approved by the institutional ethics committee (Milano Area 1 protocol number 2020/ST/298, approval date December 2, 2020); the study was performed in line with the principles of the Declaration of Helsinki. Written informed consent for participation and continuous wearable monitoring was obtained from the parents or legal guardians of all children. All data were pseudonymized before analysis, stored on secure institutional servers, and processed in full compliance with the General Data Protection Regulation (EU GDPR 2016/679). Given that the dataset includes health- and activity-related data from children (a vulnerable population), sharing individual-level data is subject to strict legal and ethical safeguards under the GDPR and the Italian Personal Data Protection Code. Owing to these restrictions and because this paper presents a tutorial and is not intended as a clinical validation, individual-level data will not be publicly shared.

### Step 3: Experimental Data Collection

The third step consists of collecting experimental data, which come not only from the wearable device but also from different sources.

In this application, 2 device models (Fitbit Charge 2 and Charge 3) were used for wearable data collection; assignment to participants was determined by device availability and was not logged at the individual level.

Personal data used in this study included age (computed in months), sex, standing height, weight, BMI, and BMI *z* score (as explained later). A Harpenden stadiometer (Holtain Ltd) with a fixed vertical backboard and an adjustable headpiece was used to measure standing height. Weight was measured with the patients standing upright at the center of the scale platform (Seca) without shoes and wearing light clothing. They faced the recorder, kept their hands at their sides, and looked straight ahead.

The clinical outcomes evaluated in this study were the 6MWT and its *z* score with respect to the normative data reported by Geiger et al [53] and the percentiles of systolic arterial pressure (SAP) and diastolic arterial pressure (DAP). The latter two were measured in the supine position using an electronic mercury sphygmomanometer (A&D Medical) with an appropriately sized cuff on the right arm after 5 minutes of rest.

### Step 4: Aim of the Research Work

As mentioned in step 2, the same dataset is generally used for more than one research aim. For this reason, a specific research project often requires further selection of the data collected that are needed to be retained for that specific aim.

Of the wearable data available in this study, only those regarding the first week of the program were selected and compared with clinical outcomes in the research work, so that the values could be considered as baseline because the effect of the exercise training program would be negligible after 1 week.

Conversely, this tutorial goes deeper into showing the opportunities that can be exploited by using the whole activity and cardiovascular dataset, without necessarily having used them in this specific research work. Additionally, the tutorial explains how to deal with anthropometric data in the case of children. Specifically, this tutorial is primarily intended for researchers, biomedical engineers, and clinicians interested in the scientific use of commercial wearables for pediatric monitoring, specifically for monitoring school-aged children. Its purpose is not to provide consumer guidance but to illustrate, step-by-step, how to extract, preprocess, and interpret wearable-derived physiological and activity data in a research context. By combining practical technical explanations (eg, data file structure and feature computation) with clinical interpretation, the tutorial aims to bridge the gap between data science and pediatric health care applications.

### Step 5: *z* Score and Percentile Data in Children

In the case of pediatric patients, it must be highlighted that parameters are generally expressed relative to normative data for a given age and sex. This is mostly expressed in terms of *z* score or percentile. The fifth step consists of adjusting all relevant parameters with respect to normative data so that children of different ages and sexes can still be compared and analyzed together.

Age was used in this study to obtain relevant reference data for the computation of *z* scores and percentiles. When the mean of the sample μ and the SD σ are made available, equation (1) is used to compute the *z* score:


(1) $$z=\frac{x-\mu }{\sigma }$$

Here, z denotes the *z* score, and x represents the observed value.

When μ is available, but not σ, z can be computed by knowing the SE of the mean and the numerosity of the sample n (ie, the number of people from which data were collected), equation (2) can be used instead:


(2) $$z=\frac{x-\mu }{SE\cdot \sqrt{n}}$$

To obtain the BMI *z* score, the BMI was then converted into its *z* scores using World Health Organization reference values [54]. In this scenario, because μ and σ are provided as reference values, equation (1) can be applied.

Resting PR (RPR) *z* scores are determined using national reference data from the 1999‐2008 US population across all age groups [55]. In this case, μ, SE, and n are available, so equation (2) is used.

The blood pressure percentile was determined for each child following recent guidelines [56-58].

### Step 6: Device Characteristics

The previously listed steps can be applied to data from all adult-designed wearables. The tutorial can also be used in the case of other devices collecting similar variables but requires more adaptations. The sixth step consists of knowing specific model characteristics and data formats, which may present slight changes even in the case of subsequent versions of the same device. It is always important to thoroughly consult all available documents and manuals before starting to analyze data.

Although some technical details of the considered devices are publicly available and downloadable from the company’s website, a concise summary is included here to guide researchers in identifying which data files and parameters are most relevant for pediatric monitoring studies. The purpose of this section is not to reproduce the device manual but to provide practical instructions on how to locate, interpret, and preprocess key Fitbit outputs for subsequent scientific analysis. Although Fitbit labels its optical measurement as HR in device interfaces and documentation, this value is in fact a PR estimate derived from a peripheral PPG sensor; the use of the term “HR” reflects commercial and user-facing conventions rather than physiological accuracy. For this reason, throughout this tutorial, we refer to this signal as PR when we talk about the physiological variable, while we maintain the original nomenclature when we refer to the file or the Fitbit-computed parameter (eg, in the case of HRZ). We refer to PR over steps as PROS and to resting PR as RPR, as previously defined.

While some parameters—such as PR and step counts—are calculated directly by the Fitbit, other metrics can be derived or estimated from the downloadable data. This section is dedicated to the parameters that can be extracted directly from the Fitbit device.

Throughout this protocol, the Fitbit Charge 2 [59] and Charge 3 [60] were used interchangeably as stated before in the manuscript. These models were selected because they have been validated previously in several peer-reviewed studies assessing the accuracy of PPG-based PR and step count estimation [61,62], ensuring data comparability with existing literature. Furthermore, they were the devices available to our research group at the time of data collection (in 2021), which allowed maintaining consistency across participants.

Although we used both the Fitbit Charge 2 and Charge 3 devices and did not perform a direct head-to-head comparison, existing independent validation studies show that the Charge 2 provides acceptable PR measurement versus ECG in 24-hour ecological recordings in adult populations (mean bias −3.47 bpm) [63], while the Charge 3 showed a larger bias (mean bias −7 bpm) but in a protocol focused on more dynamic activities when compared to a chest‐strap reference [64]. On this basis, one can assume a functional equivalence of the used models for the primary metrics (step count and PR), acknowledging that minor differences in sensor or firmware may exist. Newer Fitbit generations were not used to avoid potential discrepancies arising from firmware or algorithmic updates that could compromise data uniformity and comparability with validated models.

Both devices are equipped with a micro-electromechanical system 3-axis accelerometer to track motion patterns, an altimeter to track altitude changes, and an optical PR tracker based on PPG. As stated previously, in Fitbit documentation and app interfaces, this parameter is referred to as HR for commercial and communication purposes; however, as it is derived from peripheral PPG rather than ECG, it technically represents PR rather than HR [65].

Both devices contain a Bluetooth 4.0 radio transceiver to send data to a mobile app and can store 7 days of minute-by-minute statistics and 30 days of summary totals.

### Step 7: Import Data

The seventh step consists of a first selection of the files to be imported or discarded and of importing the files of interest for the analysis.

Different files are available for analysis for each patient, all in .json format. Data structures vary between files: some contain time series of specific parameters with associated time stamps, others contain daily recaps, and still others contain weekly recaps. Synchronization between different files was performed using the time stamps in the files that were downloaded from the Fitbit device.

**Figure 1.**
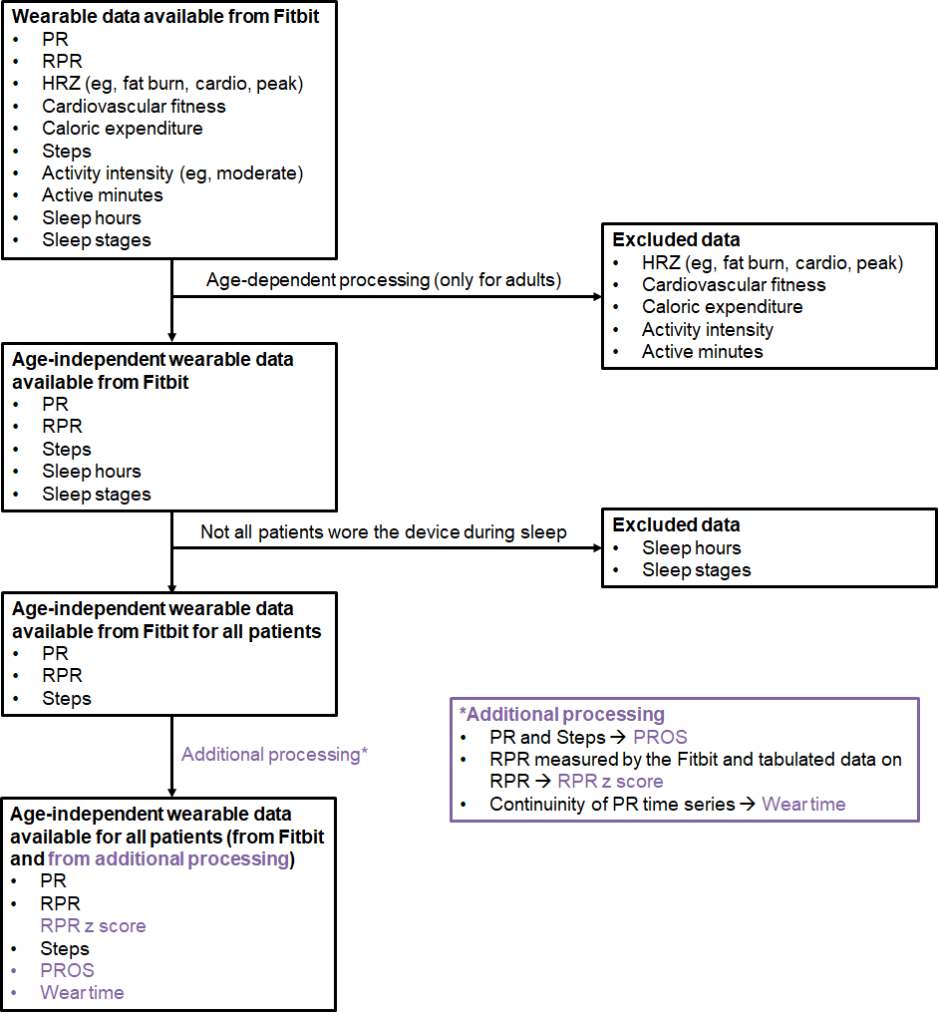
Flowchart illustrating the process from all wearable data available from Fitbit to the list of retained variables and the variables obtained with further signal processing (highlighted in purple). The purple tab is dedicated to the additional processing pipeline, that is, to explain how additional variables can be obtained. HRZ: heart rate zone; PR: pulse rate; PROS: pulse rate over steps; RPR: resting pulse rate.

Before importing the data, the first essential filtering step is required. Any file whose content depends on age-based proprietary algorithms must be excluded because Fitbit profiles must be set to 18 years or older, and several derived metrics (eg, sleep stages, sleep score, caloric expenditure, and $\dot{V}O_{2}$ max–related fitness scores) are computed using adult-specific thresholds and models. For this reason, files related to sleep analysis, HRZ, calories, or fitness level should be discarded, as their values would be systematically biased in a pediatric cohort. More details regarding discarded metrics are reported in Figure 1.

Providing a fully reproducible data import script is not useful to readers because Fitbit intraday files do not follow a fixed header structure; field names differ across device models, firmware versions, application programming interface generations and types (eg, consumer vs research interface), and, more recently, Google-managed data export formats, requiring manual inspection and adjustment at import. More details on this can be found in the Data Import section of Multimedia Appendix 1.

### Step 8: Activity Metrics

The eighth step consists of exploring files with parameters related to the activity and selecting age-independent activity-related metrics.

#### “Activities” File and Derived

The “activities” file contains a daily summary of Fitbit activity data and goals, covering metrics such as steps, floors, active minutes, distance, and calories burned. The main fields include “activities,” “goals,” and “summary.”

Specifically, the field “activities” is an array listing any detailed logged activities. If this array is empty, this indicates that there were no manually logged activities on that day.

The field “goals” describes the user’s target goal for the day in terms of “activeMinutes” (target active minutes), “caloriesOut” (target calories to be burned), “distance” (target distance), “floors” (target floors), and “steps” (target steps).

The field “summary” reports the actual daily data, specifically “activeScore” (an internal scoring metric used by Fitbit), “activityCalories” (calories burned during active minutes), “caloriesBMR” (basal metabolic rate—calories burned at rest), “caloriesOut” (total calories burned, including basal metabolic rate and activity), “distances” (an array of distance values broken down by activity levels), “elevation” (floors climbed in terms of elevation gain; may be 0 if not measured), “fairlyActiveMinutes” (minutes of moderate activity), “floors” (floors climbed), “heartRateZones” (an array showing the time spent in each HRZ—out of range, fat burn, cardio, and peak—along with estimated calories burned in each zone), “lightlyActiveMinutes” (minutes of light activity), “marginalCalories” (extra calories burned while being lightly active), “sedentaryMinutes” (minutes spent in a sedentary state), “steps” (total number of steps taken), and “veryActiveMinutes” (minutes of very active exercise).

All the fields regarding the count of calories (“activeCalories,” “caloriesOut,” “caloriesBMR,” and “marginalCalories”), as well as the “calories” file, contain estimations of caloric consumption that are likely based on age, sex, weight, and height used as input in the Fitbit app. As the Fitbit device is designed for adults, it was only possible to set the age to 18 years or older to have the app working. For this reason, these metrics cannot be considered reliable in the present application, as already mentioned in step 7. The weekly recap files associated with these variables must also be excluded from the analysis.

The same can be said for the information related to the intensity of the activities based on HRZ values, which are still the subject of research in adults and are computed considering the maximum HR for a given age (which was set as 18 y in the application). In addition, in this case, the weekly recaps have been excluded.

For this reason, the only reliable metric is “steps.” The number of walked steps is computed using the 3-axis accelerometer embedded in the Fitbit with dedicated processing algorithms. A traditional threshold of 10,000 daily steps is well accepted as indicative of an active lifestyle in adults [66]. To evaluate patient performance, one can consider either the mean daily steps or the maximum daily steps, representing the highest number of steps achieved during the test period. The latter is more representative of the ability to walk a certain distance [67].

**Figure 2.**
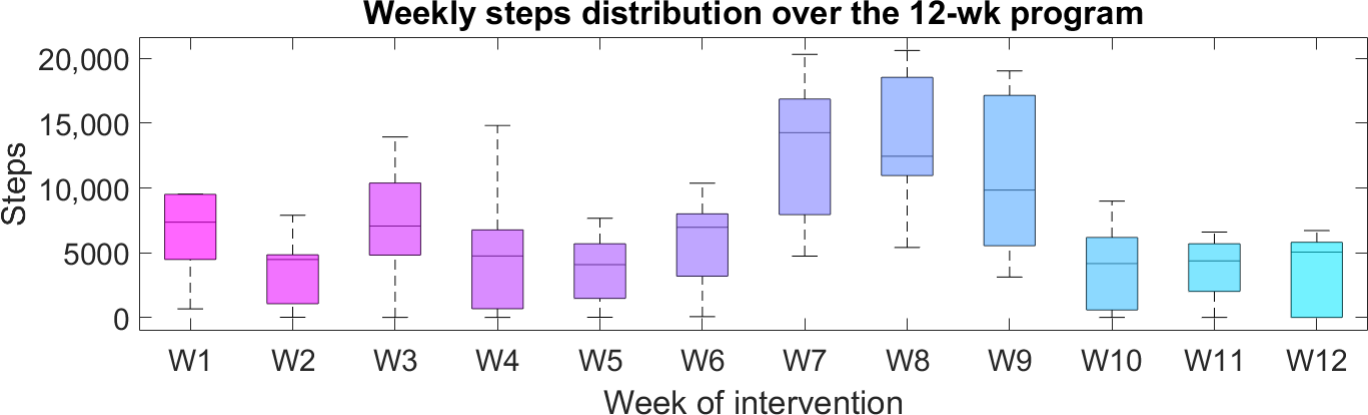
Weekly steps distribution over the 12-wk online exercise training program (boy, aged 10 y; BMI=23.7 kg/m^2^; BMI *z* score=1.74).

The file “range_activities_steps” reports the total number of daily steps for a week. Visualizing this information, extracted either from the daily metrics or from the weekly recap files, can be used to immediately visualize behavioral shifts of patients. Figure 2 shows the distribution of daily steps in the 12 weeks of the intervention in the case of one patient. The higher step counts observed during weeks 7 to 9 likely coincide with the central weeks of August, when many families in Italy are on summer vacation, possibly leading to increased physical activity; however, this interpretation remains speculative.

#### “Steps” File

**Figure 3.**
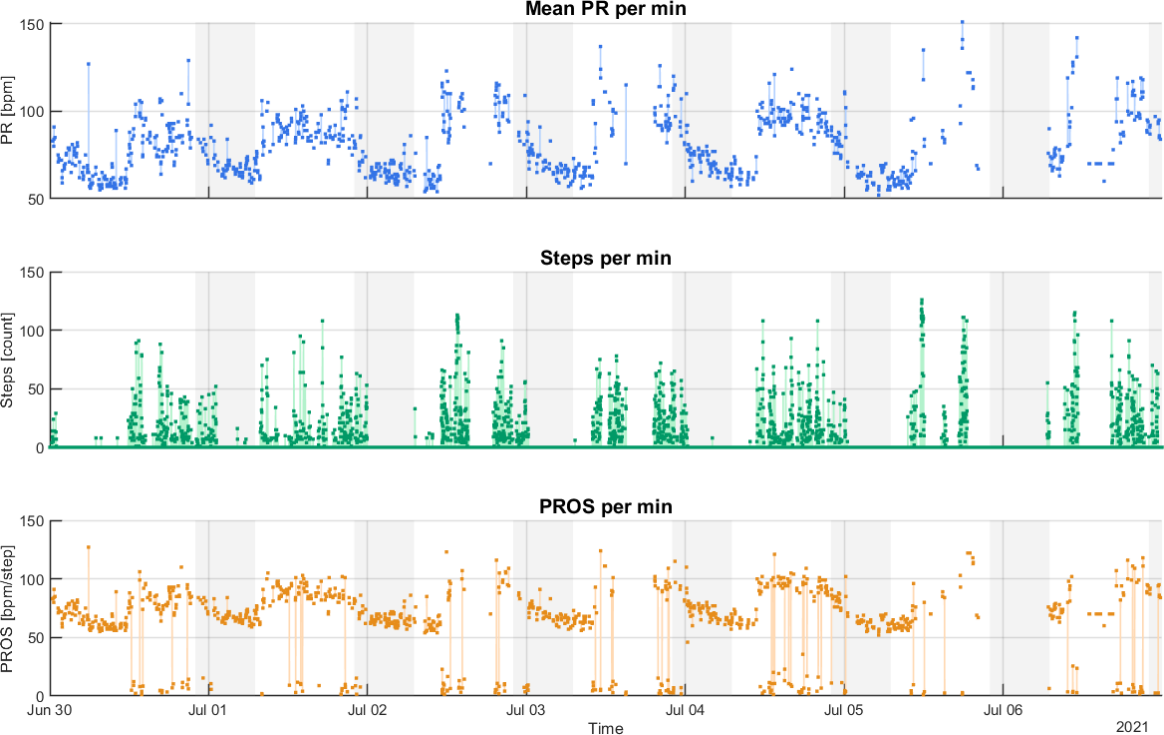
Example of pulse rate (PR), steps, and pulse rate over steps (PROS) obtained in 1 wk of acquisitions (boy, aged 12 y; BMI=27.1 kg/m^2^; BMI *z* score=1.86). Areas with the gray background correspond to night hours (22:00-06:59), while areas with the white background correspond to day hours (07:00-21:59). bpm: beats per minute.

Unlike the steps derived from the “activities” files family, the daily “steps” file shows the distribution of the steps during each day, in addition to the total number of steps. This allows to see when steps were taken during the day and better track the times of day when there has been the most activity. Examples of this can be seen in Figure 3.

In the considered clinical case example, there were 3 cases when an overall daily step count was provided, but no data on the distribution of the steps. This lack of detailed information on the distribution prevents the computation of other parameters (PROS, explained in a subsequent section).

### Step 9: Cardiovascular Metrics

The ninth step consists of exploring files with parameters related to the cardiovascular metrics and selecting age-independent metrics. As was mentioned earlier, some data, such as RPR, need to be adjusted for age and sex even if acquired from the wearable devices; in all relevant cases, step 5 should be repeated.

PR is continuously estimated via the embedded PPG sensor, with the raw data processed by an internal proprietary algorithm [67].

#### “hr” File

The daily “hr” file contains information regarding the already processed PR. There is an array (“activities-heart”) containing the summary of the daily information, and then there is the “activities-heart-intraday” array with more details. This array has “dataset” as the primary subfield, which is an array of time-stamped entries; each entry has time and value. Additionally, there are 2 metadata fields, “datasetInterval” and “datasetType,” which describe how the intraday data are structured.

PR measurement is affected by the location of the device and movement, as both factors can alter optical coupling, blood perfusion, and motion-induced artifacts, leading to transient inaccuracies or missing data—limitations common to all wrist-based PPG systems [68]. Furthermore, data are available at different sampling rates, with an interval in the order of seconds, one from the other at rest, and an increased sampling rate when exercise is detected. RPR is calculated over a 24-hour period and is known from the literature to reach its lowest value at night [69]. Consequently, nighttime RPR values are directly comparable across patients, whereas daytime values may not reflect the true minimum.

PR variability (PRV) is highly correlated with the HR variability, which quantifies the extent to which the R-R interval or HR changes from one cardiac cycle to the next. This measure can provide a lot of information about how the autonomic nervous system regulates the heart. HR variability has been correlated with cardiac diseases [70], fitness, and functional reserve [71] and is positively affected by exercise [72]. During our study, no PRV values were made available for download, nor could they be derived as no raw signal was given. As we reported in a previous study [67], PR data retrieved from a Fitbit device presented considerable gaps in the recordings, even for continuous recordings. As stated by the manufacturer, the normal sampling period should be between 5 and 15 seconds; however, in the results of that study, lack of data for 15 seconds or more was common. This makes it challenging to compute PRV reliably, so this metric should be analyzed with caution if obtained from this type of device.

Fitbit Charge 2 and 3 also provide information regarding HRZ and consequently the number of minutes spent in each zone. In adults, these data can be used to estimate the physical activity level of the person. However, in the present pediatric application, their reliability cannot be verified because the underlying proprietary algorithms were developed and validated on adult populations, and their exact computation methods are not publicly available.

### Step 10: Combining Activity and Cardiovascular Metrics

The tenth step consists of combining activity and cardiovascular metrics. The parameters computed in this work were selected based on the clinical case and research aims, but other parameters can be considered as well.

Combining the metrics obtained from the activity and the cardiovascular domain is possible, and one example is reported in this tutorial. Mishra et al [73] used a Fitbit device to detect SARS-CoV-2 infection before symptom onset. To achieve this, the authors introduced and calculated the HR over steps index to identify potential anomalies in cardiac response relative to physical activity. In this study, this index is referred to as PROS to maintain homogeneity with the rest of the nomenclature.

PROS is computed as the instantaneous ratio between PR and the number of steps recorded within the corresponding time window. As PR is sampled approximately every 5 seconds and steps are aggregated per minute, each minute-level step count was matched to all PR values recorded within that same minute. To avoid division by zero during sedentary periods, 1 step was added to the denominator. The resulting value can therefore be expressed as in equation (3):


(3) $$PROS\left(t\right)=\frac{PR\left(t\right)}{steps\left(t\right)+1}$$

where PR(*t*) represents PR at time *t* (beats per minute) and steps(*t*) is the number of steps detected within that minute. PROS provides a minute-by-minute estimate of the patient’s cardiovascular effort and functional capacity. A high PROS value indicates a rapid heartbeat during moderate physical activity, while a low value suggests a restrained heartbeat despite challenging physical activity.

Mishra et al [73] developed a structured algorithm for computing PROS and identifying anomalous values, leveraging a dispersion-based approach that detects deviations from a normal distribution using the elliptic envelope method. The code used to process the data was retrieved from the GitHub repository associated with the original study. The anomalies are detected using a contamination of 0.1, where the contamination means the number of outliers in the dataset. In this manner, the algorithm identifies some outliers in the computed feature, corresponding to approximately 10% of the total. Following the method in the original paper, an iterative approach was used, incrementally increasing contamination values until a stable number of outliers was identified.

A possibility is to evaluate PROS at low quantiles, such as the 1% or the 5% quantile, to account for the possibility that the minimum PROS is an outlier.

As was mentioned before in the section dedicated to steps, it was not possible to compute PROS in 3 patients, as Fitbit provided the overall step count but not the details of the distribution of the steps during the day.

### Step 11: Data Quality Assessment

The eleventh step of this tutorial consists of assessing the quality of the collected data, especially in terms of completeness of the dataset. Wearable data collection is unsupervised, and for this reason, patient adherence to the data collection protocol, that is, wear time, must be carefully evaluated in each study. In addition, signal loss is an important parameter to be evaluated, as explained later in the section.

To assess signal completeness and compliance, wear time can be quantified based on PR data continuity. In this dataset, the time intervals between consecutive PR samples were computed for each participant; intervals of 2 minutes or less were considered “worn,” while longer gaps indicated device removal. Wear time was then expressed as the total duration of “worn” periods divided by the total duration of the analyzed time window. The 2-minute PR gap threshold was selected as a conservative compromise, as short interruptions of a few seconds are common even when the device is correctly worn. Adult Fitbit studies have proposed different definitions: Dominick et al [74] considered 60 minutes or more of missing signal as nonwear; Orstad et al [75] allowed up to 90 minutes of interruption to consider a day valid (with ≥10 h of recording including the interruption); Claudel et al [76] used minute-level PR gaps to classify nonwear episodes. Given the higher motion and frequent repositioning typical of children, the 2-minute criterion minimizes false nonwear detection while maintaining sensitivity to genuine removal periods.

To quantify how this threshold influenced data retention, it is advised to perform a sensitivity analysis. We performed such calculations using all 16 participants and recalculated wear time with nonwear gap thresholds of 60, 90, 120, 180, and 300 seconds. Weekly wear time changed only minimally across thresholds (60 s: 100.8 h; 90 s: 100.9 h; 120 s: 101.1 h; 180 s: 101.4 h; and 300 s: 101.8 h), with differences never exceeding 1.0 hour per week on average. Similarly, the percentage of days achieving 70% or greater completeness remained constant at 41% for all thresholds. These results indicate that the 120-second rule does not artificially inflate wear time estimates and that more permissive thresholds (eg, 180‐300 s) offer no meaningful advantage in terms of data retention in this cohort. Day and night were defined as 07:00 to 21:59 and 22:00 to 06:59, respectively, to align with typical waking and sleeping schedules in school-aged children. The theoretical acquisition window corresponded to 168 hours per week, split into approximately 105 hours daytime and 63 hours nighttime.

In addition to wear time, a second essential component of data quality is the assessment of signal loss, which reflects gaps in acquisitions even when the device is being worn. Unlike nonwear episodes, signal loss is typically caused by transient motion artifacts, insufficient optical coupling, or abrupt changes in perfusion—phenomena that are especially common in children due to rapid, irregular movements. To quantify signal loss, it is advised to examine the distribution of intersample intervals across the PR time series and identify gaps that exceed the expected sampling pattern (typically 5‐15 s at rest, with denser sampling during activity). These gaps should be flagged as within-wear missing segments, which differ clinically and analytically from nonwear periods. For example, brief clusters of missing samples may be acceptable when computing aggregated daily features (eg, daily steps and mean PR) but can severely affect features that rely on continuous sampling, such as PROS, nocturnal minima, or any attempt to estimate PR variability. Figure 1 reports a flow diagram of the Fitbit-derived metrics retained or excluded from the analysis, the reasons for exclusion, a list of the metrics that have been computed with additional processing, and a tab dedicated to how additional processing is performed for each variable.

### Step 12: Statistical Analysis

The final step is the statistical analysis and is again generalizable like steps 1 to 5. First, traditional statistical metrics such as mean and SD should be computed for each parameter, possibly further divided into subpopulations as done in Table 1, to characterize the population. Then, further steps of statistical analysis must be tailored to the specifics of the study.

In this clinical case, correlation analyses were performed to obtain the Pearson correlation coefficient ρ, the coefficient of determination *R*^2^, and the *P* value; then a multiple linear regression analysis [77] was conducted to predict the 6 clinical outcomes listed in Textbox 1. Both the raw resting RPR and its age- and sex-adjusted *z* score were included as predictors in the regression models. Although these variables are mathematically related, they were retained together to explore whether absolute RPR and its standardized deviation from pediatric norms contribute differently to the prediction of functional outcomes. This approach was adopted intentionally to capture both absolute physiological values and relative, age-adjusted variability, acknowledging that some degree of correlation between the two variables may occur.

To mitigate overfitting due to the small sample size, an additional model selection analysis was conducted using leave-one-out cross-validation (LOOCV) combined with least absolute shrinkage and selection operator (LASSO) regularization. This approach automatically shrinks or removes redundant predictors, allowing a more parsimonious set of variables to emerge for each clinical outcome. The models were then refitted using only the selected predictors to verify their explanatory power.

**Table 1.** Summary of demographic, cardiovascular, and physical activity metrics of the whole cohort (N=16) and then divided by boys (n_boys_=11) and girls (n_girls_=5).

Parameter	Overall (N=16), mean (SD)	Boys (n_boys_=11), mean (SD)	Girls (n_girls_=5), mean (SD)
Age (y)	10.2 (1.7)	10.2 (1.3)	10.2 (2.6)
BMI (kg/m²)	28.1 (3.7)	27.1 (3.0)	30.2 (4.5)
BMI *z* score	2.2 (0.3)	2.1 (0.3)	2.4 (0.4)
RPR^a^ (bpm)	79.4 (9.4)	79.3 (8.9)	79.5 (11.3)
RPR *z* score	−0.06 (0.45)	−0.03 (0.47)	−0.15 (0.42)
SAP^b^ (mm Hg)	111.7 (11.1)	109.9 (12.6)	116.5 (2.4)
SAP percentile (%)	76 (27)	72 (30)	88 (8)
DAP^c^ (mm Hg)	73.1 (8)	71.5 (8)	77.5 (7)
DAP percentile (%)	82 (16)	79 (17)	91 (10)
Daily steps	7742 (3025)	7838 (3629)	7530 (1148)
Maximum daily steps	12,895 (4964)	13,279 (5873)	12,050 (2210)
6MWT^d^ distance (m)	490.5 (55.0)	487.2 (55.6)	517.0 (50.4)
6MWT z score	−2.7 (1.1)	−3.0 (0.9)	−2.0 (1.3)
Total wear time (weekly) (h)	101.1 (44.6)	105.5 (43.8)	91.4 (49.8)
Total wear time (weekly) (%)	60.2 (26.5)	62.8 (26.1)	54.4 (29.6)
Day wear time (weekly) (h)	72.3 (27.3)	73.8 (22.9)	68.8 (38.2)
Day wear time (weekly) (%)	68.8 (26.0)	70.3 (21.8)	65.5 (36.4)
Night wear time (weekly) (h)	28.8 (23.0)	31.6 (23.6)	22.6 (22.8)
Night wear time (weekly) (%)	45.7 (36.5)	50.2 (37.4)	35.9 (36.2)

a RPR: resting pulse rate

b SAP: systolic arterial pressure

c DAP: diastolic arterial pressure

d 6MWT: 6-minute walk test

Textbox 1. **Input data used for the analysis and the clinical outcomes of the presented clinical application.**

Input data

- Personal data (anthropometric)

  -  Age
  -  BMI
  -  BMI *z* score

- Wearable data (week 1)

  -  6-minute walk test
  -  6-minute walk test *z* score
  -  Systolic arterial pressure
  -  Systolic arterial pressure percentile
  -  Diastolic arterial pressure
  -  Diastolic arterial pressure percentile

Clinical outcomes

- Mean daily steps
- Maximum daily steps
- Resting pulse rate
- Resting pulse rate *z* score
- Pulse rate over steps

## Results of the Considered Clinical Case

### Population Characteristics

A summary of demographic, cardiovascular, and physical activity metrics for the cohort is presented in Table 1, including data divided by boys and girls. These values are expressed in terms of mean (SD).

Quantitative wear time statistics per participant can be reported in greater detail, as proposed in Table S1 in Multimedia Appendix 1.

### Collected Data

An example of time series plots of PR, steps, and PROS obtained in a week of acquisitions is shown in Figure 3.

**Figure 4.**
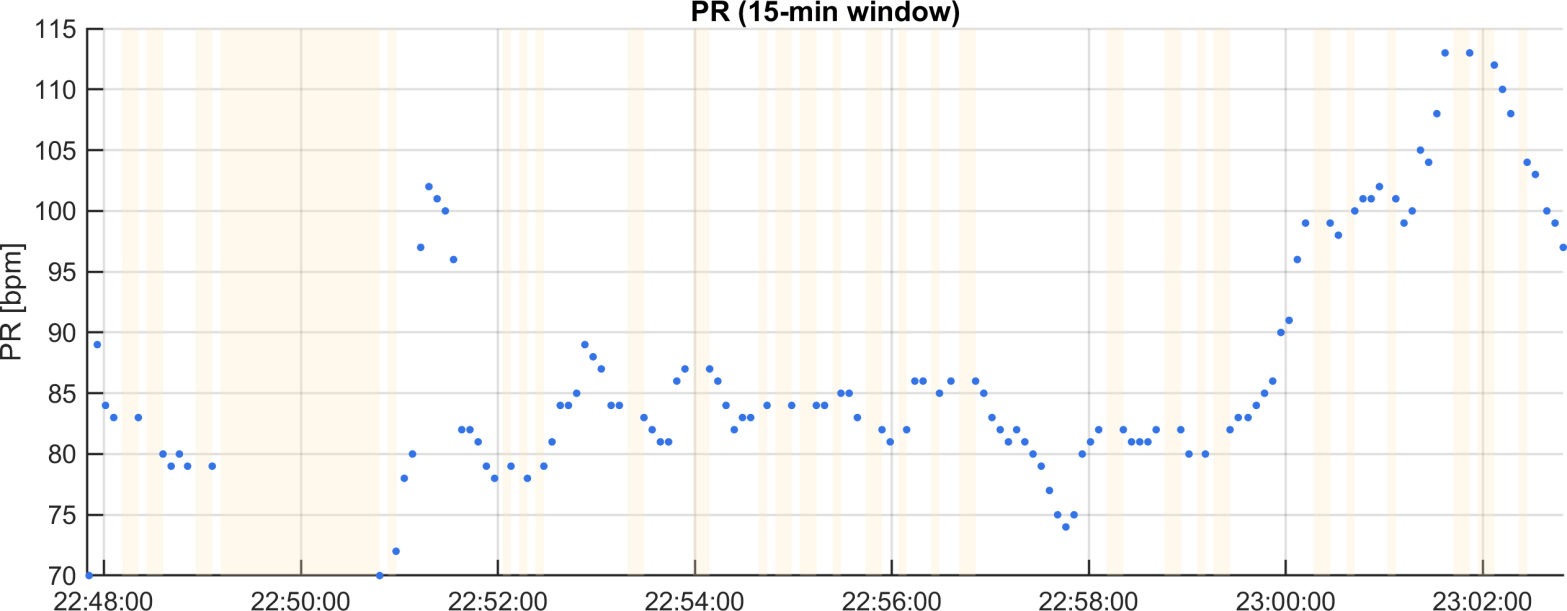
Representative 15-min window of PR data from the same participant as in Figure 3 (boy, aged 12 y; BMI=27.1 kg/m^2^, BMI *z* score=1.86). Blue dots represent individual PR samples, while light orange shaded areas indicate missing intervals where the time between consecutive samples exceeded 1.5×the expected sampling period. bpm: beats per minute.

To further illustrate the temporal pattern of PR recordings and the occurrence of signal loss, Figure 4 shows a representative 15-minute segment extracted from the same week of acquisition plotted in Figure 3. This high-resolution view highlights the irregular sampling of PR data and the short interruptions occurring when the signal was not acquired by the Fitbit device.

### Statistical Analysis

Table 2 reports Pearson correlation coefficients (ρ) to explore the relationships among wearable data and clinical outcomes.

It is noteworthy that RPR *z* score has a significant correlation with 6MWT results (ρ=−0.53; *R*^2^=0.28; *P*=.04), and it is a negative correlation as is expected from physiology. These results directly show the utility of wearable-derived data in clinical outcome assessment.

Two additional statistically significant correlations are observed between maximum weekly steps and the 6MWT (ρ=−0.52; *R*^2^=0.27; *P*=.04) and between maximum weekly steps and the 6MWT *z* score (ρ=−0.60; *R*^2^=0.36; *P*=.01); however, such results are not what one would intuitively expect, nor do they confirm what was obtained in a population of older patients [67]; therefore, it is likely a spurious correlation in both cases, as is discussed in more detail later in the paper.

The mean daily steps showed a moderate negative correlation with the DAP percentile (ρ=−0.42; R^2^=0.18; *P*=.12) but did not reach statistical significance.

PROS displayed only weak correlations with functional capacity measures, indicating that this variable alone might have limited predictive value in this context. RPR and RPR *z* score showed a moderate, nonsignificant correlation with DAP (ρ=0.41; *R*^2^=0.17; *P*=.13; and ρ=0.43; *R*^2^=0.19; *P*=.12, respectively), and the RPR *z* score also showed a moderate correlation with DAP percentile (ρ=0.42; *R*^2^=0.17; *P*=.14).

**Figure 5.**
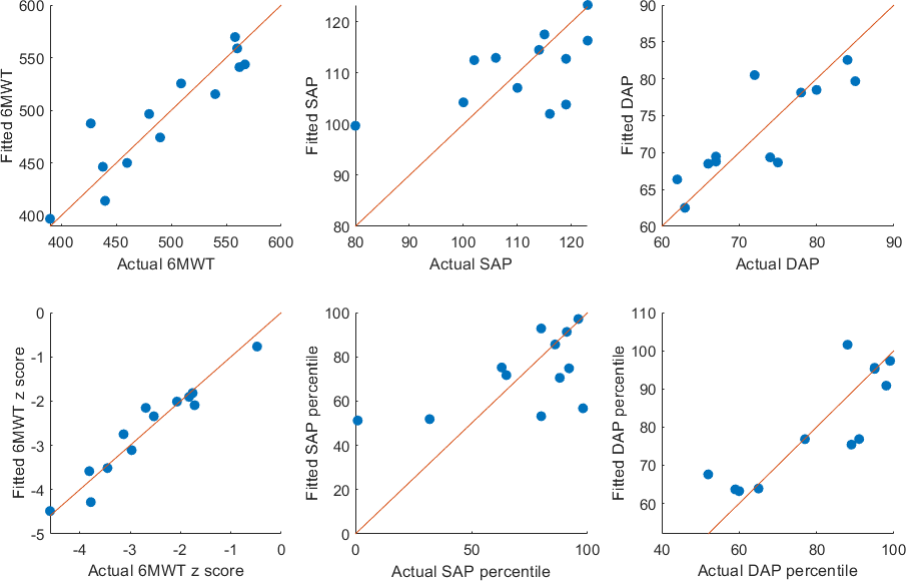
Observed versus fitted clinical outcomes (6-min walk test [6MWT], 6MWT *z* score, systolic arterial pressure [SAP], SAP percentile, diastolic arterial pressure [DAP], and DAP percentile). The scatter plot illustrates the relationship between observed and fitted distances, with the red dashed line indicating an ideal fit.

Multiple linear regression models were constructed to examine the associations between wearable data and clinical outcomes. Figure 5 shows the observed and the fitted values of each of the variables, with statistical significance defined as *P*<.05.

with The regression model for fitting 6MWT distance yielded an adjusted *R*² of 0.51, indicating a moderate fit of the model; the regression model fitting the 6MWT *z* score had a stronger fit (adjusted *R*²=0.79), and, overall, the best performance among the 6 clinical outcomes considered in the study. In this case, 2 variables, that is, RPR and its *z* score, made a statistically significant contribution to the model (*P*=.03 and *P*=.02, respectively), indicating the strong dependence of the 6MWT *z* score on parameters relating to cardiac activity.

SAP, SAP percentile, DAP, and DAP performed poorly in the case of adjusted *R*^2^, with the value being negative because there are too many variables. Given the small cohort size (n=16), all regression models should be considered underpowered; therefore, their estimates are intended to be illustrative of the methodological workflow and not to provide confirmatory inference. Still, all predictors in the full multivariable models, with coefficient, SE, *P* value, *R*^2^, adjusted *R*^2^, and n, are reported for each outcome in Table S2 of Multimedia Appendix 1.

The LASSO procedure identified age, BMI, maximum daily steps, and RPR *z* score as predictors of the 6MWT distance (adjusted *R*²=0.29) and age, BMI *z* score, mean and maximum daily steps, RPR, RPR *z* score, and PROS for the 6MWT *z* score (adjusted *R*²=0.75). Details on the predictors selected by LASSO with LOOCV for each outcome are reported in Table S3 of Multimedia Appendix 1. For blood pressure–related outcomes, the regularization excluded most predictors, retaining only maximum daily steps for systolic measures and BMI for diastolic pressure, both with negligible explanatory value (adjusted *R*²≈0).

**Table 2.** Correlation of wearable data with clinical outcomes.

Variable	Clinical outcomes																	
	SAP^a^			SAP percentile			DAP^b^			DAP percentile			6MWT^c^			6MWT z score		
	ρ	*R* ^2^	*P* value	ρ	*R* ^2^	*P* value	ρ	*R* ^2^	*P* value	ρ	*R* ^2^	*P* value	ρ	*R* ^2^	*P* value	ρ	*R* ^2^	*P* value
Wearable data																		
Mean steps	−0.04	0.00	.90	−0.13	0.02	.64	−0.22	0.05	.44	−0.42	0.18	.12	0.07	0.01	.79	0.05	0.00	.85
Maximum steps	0.12	0.01	.67	0.14	0.02	.63	0.20	0.04	.47	0.16	0.03	.56	−0.52	0.27	*.04^d^*	−0.60	0.36	.01*^d^*
RPR^e^	0.15	0.03	.57	0.12	0.01	.67	0.41	0.17	.13	0.35	.12	.21	−0.38	0.14	.15	0.03	0.00	.90
RPR z score	0.24	0.06	.40	0.23	0.06	.44	0.43	0.19	.12	0.42	0.17	.14	*−0.53*	*0.28*	*.04^d^*	−0.34	0.12	.21
PROS^f^	−0.14	0.02	.66	−0.17	0.03	.59	0.08	0.01	.81	0.20	0.04	.52	0.20	0.04	.52	0.21	0.04	.49

a SAP: systolic arterial pressure.

b DAP: diastolic arterial pressure.

c 6MWT: 6-min walk test.

d Statistically significant correlations.

e RPR: resting pulse rate.

f PROS: pulse rate over steps.

These results confirm that, in this limited cohort, 6MWT and 6MWT *z* score remain the only variables moderately predictable from wearable-derived metrics, while SAP, SAP percentile, DAP, and DAP percentile show minimal dependence on these predictors. The analysis demonstrates that the negative adjusted *R*² values observed in the unregularized multiple regressions shown in Figure 5 are primarily due to sample size limitations rather than methodological flaws.

## Discussion

This tutorial explains step by step how to process and further engineer activity and cardiovascular data obtained from Fitbit when used in pediatric patients and presents a clinical application in children with obesity.

### Study-Specific Findings

The data gathered from Fitbit Charge 2 and Charge 3 in children with obesity demonstrate that consumer-grade wearable devices can provide clinically relevant information about physical activity (eg, mean or maximum daily steps) and cardiovascular function (eg, RPR and PROS) in this population. As shown, these metrics alone do not exhibit strong correlations with standard reference tests (eg, the 6MWT in children or systolic or diastolic blood pressure percentiles). However, by supplementing them with anthropometric indicators and appropriate pediatric reference values, a more robust framework can be built for understanding functional capacity and exercise tolerance. Such results are also expected to improve with larger datasets available. Among the observed statistically significant associations, there were inverse correlations between maximum daily steps and both the 6MWT distance and its *z* score. These associations should be interpreted as exploratory, as the analysis is constrained by the small sample size (n=16), which limits statistical power and the generalizability of the findings. Additionally, the counterintuitive finding of having inverse correlations may have several explanations. First, given the small sample size and multiple comparisons, a spurious correlation cannot be excluded. Second, the 6MWT reflects submaximal exercise tolerance measured under clinical supervision, whereas maximum daily step counts reflect spontaneous physical activity in free-living conditions. Children with lower functional capacity may have been more engaged to increase their daily steps during the monitoring week, potentially due to parental encouragement after clinical evaluation. Measurement variability may also contribute: step count accuracy in children can be affected by shorter stride length, irregular gait, and differences in device placement, leading to over- or under-counting at certain speeds. Additionally, the week chosen for wearable data collection may not coincide with typical activity patterns—for example, periods of school holidays or illness could bias step count data independently of actual fitness.

Overall, our findings indicate that consumer-grade wearable devices, such as the Fitbit Charge 2 and Charge 3, can provide meaningful information on both physical activity and cardiovascular parameters in children with obesity. These results suggest a potential role for such devices as complementary tools in clinical and research settings to monitor behavior and physiological responses outside the laboratory environment, hence the need to provide practical guidelines and indications on possible data processing pipelines.

An additional limitation of our work stems from the fact that the Fitbit app enforces a minimum user age of 18 years or older, which inevitably introduces bias in the estimation of HRZ and caloric output and led us to discard such parameters. As the algorithmic assignment of HRZ is age-based (often through formulas such as “220 − age”), pediatric users are forced to adopt adult parameterization. Consequently, derived metrics such as time in “cardio” or “peak” zones and estimated energy expenditure are likely to be inaccurate when applied to cohorts of school-aged children. Further complicating matters, children’s basal metabolic rates and activity energy expenditure differ systematically from adult models (eg, children exhibit relatively higher mass-specific metabolic rates) [78]. Thus, caloric estimates based on adult-tuned algorithms may misrepresent true energy usage in minors.

Setting all profiles to 18 years or older affects multiple Fitbit-derived metrics beyond calories. Cardiovascular fitness score (a proxy of V̇O_2_ max) is explicitly age-dependent and sex-dependent [79], so values in minors will be misestimated. Finally, activity classification and intensity inference are trained on adult patterns or rely on adult cutpoints [80]; studies show poor transfer to children and wide variability when applying adult or nonpediatric thresholds, motivating pediatric-specific models [81]. In practical terms, these biases limit the validity of continuous cardiovascular monitoring using standard Fitbit commercial pipelines in children: improvements or deteriorations in cardiovascular load may be masked or exaggerated.

Some issues appear easier to overcome than others; for instance, the lack of PRV data and the presence of inconsistencies in the sampling could be dealt with by using Fitbit smartwatches instead of activity trackers. It is, in fact, possible to design custom apps for Fitbit smartwatches, but not for trackers, by exploiting the software development kit available. Nevertheless, our experience suggests that, when used in a controlled protocol and combined with rigorous data cleaning, even simple trackers can yield reliable step counts and consistent PR data—even in a cohort of children.

A key limitation of this study is the small sample size (n=16), which substantially restricts the statistical power of correlation and regression analyses. With multiple predictors included in the models, the degrees of freedom are limited, increasing the likelihood of overfitting and unstable coefficient estimates. Accordingly, the regression outputs presented here primarily demonstrate the feasibility of integrating wearable-derived features into statistical models, rather than serving as robust or generalizable predictors. Owing to this, the absence of significant associations should be interpreted cautiously, as these results may reflect insufficient power rather than a true lack of relationship between wearable-derived and clinical variables. In addition, the negative adjusted *R*² values observed in several models, together with the presence of multicollinearity among personal and wearable data, indicate that the LASSO and LOOCV results should not be overinterpreted. With such a small sample and correlated predictors, penalized regression can select unstable subsets of variables, and cross-validation may provide overly optimistic estimates of predictive value. Future studies with larger, more heterogeneous samples will be required to confirm these findings, refine the regression models, and enhance generalizability.

While data acquisition from the Fitbit devices was successful in this cohort, this analysis did not assess broader aspects of feasibility, such as long-term adherence, cost, or regulatory considerations, nor are such aspects the focus of this tutorial.

### General Considerations

The 12 steps of the tutorial can be adapted to other studies with Fitbit devices with minimal adjustments and with other devices by maintaining some of the steps (steps 1-5 and step 12) and adapting the remaining steps to the specific file format and available data. This pipeline could be easily replicated in other studies using similar devices. In a previous study conducted by our research group [67], we enrolled 31 older patients (mean age 76.1 years) to assess whether parameters that could be obtained from a Fitbit Inspire 2 device were correlated with 6MWT results. We obtained statistically significant correlation in the cases of nonexercise testing cardiorespiratory fitness (ρ=0.68; *P*<.001), RPR (ρ=−0.39; *P*=.03), HR over steps at 1% quantile (ρ=−0.39; *P*=.04), mean number of steps (ρ=0.59; *P*<.001), logarithmic transformation of the mean number of steps (ρ=0.56; *P*=.001), and V̇O_2_ max (ρ=0.58; *P*=.006).

On the basis of our findings across multiple studies, data from commercial wearable devices could offer a low-cost alternative to in-hospital assessments such as the 6MWT. This approach is particularly advantageous for patients who live far from the hospital or when hospital access is restricted, as was the case during the recent COVID-19 pandemic. Furthermore, the presented technology allows tracking one’s progress over time. Results obtained in children demonstrate that even models designed for adults could be useful; however, further considerations should be applied when devices are used on children, particularly for company-provided metrics that are not immediately connected to the raw values of parameters.

Fitbit Charge 2 and Charge 3 are low-cost, basic trackers. More recent models (eg, Fitbit Charge 6) can be purchased with less than €150 (about US $173) per unit. An advantage of such devices is that they are widespread, specifically among young people and thus already present in a large part of the population, constituting a mine of data that is constantly supplied with even more data. Beyond childhood obesity, wrist-worn PR activity workflows have relevance across multiple clinical domains. In congenital heart disease, an American Heart Association science advisory supports wearable devices for continuous home monitoring and outlines clinical, ethical, and integration considerations [82]; pediatric studies also show consumer smartwatches (in this case, the Apple Watch) can capture clinically relevant arrhythmias not detected by standard ambulatory monitors [83]. In respiratory disease, cystic fibrosis cohorts have derived individualized PR thresholds from activity trackers (the so-called HRZ) to quantify moderate-to-vigorous activity [84], while asthma programs using multimodal wearable home monitoring have shown feasibility for assessing compliance outside the clinic [85]. These represent only a few examples of possible clinical applications of wearables in school-aged children.

The main limitations of currently available commercial devices are that they generally do not provide raw data, and their metrics (such as the number of minutes of an activity of a given intensity) are computed based on adult data and a black box, generally artificial intelligence-based [86] model from the point of view of the end user. This makes it difficult, if not impossible, to understand how the algorithm was designed and on which assumptions it is based, particularly because private companies do not disclose their algorithms. If this lack of transparency could still be acceptable in the case of adult populations, where the assumption holds that substantial amounts of data have been collected in the past by major companies, this is no longer true in the case of pediatric applications.

As noted in the state-of-the-art section, most commercial devices for children are focused on activity metrics, but there are no devices that perform cardiovascular measurements. Both raw data and dedicated studies on continuous monitoring of cardiovascular parameters would enhance our ability to evaluate data quality and accuracy.

### Conclusions

This tutorial primarily aims to establish the methodological feasibility of using adult-oriented wearable devices for pediatric monitoring, rather than to test clinical hypotheses or generate clinical conclusions. Our findings show that, when appropriate preprocessing and data quality assessments are applied, low-cost wrist-worn wearables can provide some usable data in school-aged children. The clinical example illustrates how such methodological steps translate into practice, but the interpretation remains exploratory and is not intended as a validation study. Although consumer devices may not offer raw signals or entirely pediatric-calibrated metrics, the overall experience we describe indicates that step counting and PR data can still be leveraged in clinical or research contexts involving children. By applying careful methods to manage and analyze the data, clinicians and investigators can minimize inaccuracies and still benefit from remote monitoring capabilities.

To perform reliable clinical trials using wearables on the pediatric population, activity trackers have demonstrated their performance, while further research is needed to assess the accuracy of cardiovascular parameters. The methodology proposed in this tutorial, however, gives indications on how to process such data with caution and maintaining scientific rigor, which is necessary for applications in clinical trials.

## Supplementary material

10.2196/76166 Multimedia Appendix 1 Data import and supplementary tables.

## Abbreviations

6MWT: 6-minute walk test
DAP: diastolic arterial pressure
ECG: electrocardiography
GAI: generative artificial intelligence
GAIDeT: Generative Artificial Intelligence Delegation Taxonomy
HR: heart rate
HRZ: heart rate zone
ICC: intraclass correlation coefficient
LASSO: least absolute shrinkage and selection operator
LOOCV: Leave-one-out cross-validation
PPG: photoplethysmography
PR: pulse rate
PROS: pulse rate over steps
RPR: resting pulse rate
SAP: systolic arterial pressure
