# Supplementary material for: Using an Adult-Designed Wearable for Pediatric Monitoring: Practical Tutorial and Application in School-Aged Children With Obesity
Source: J Med Internet Res. 2026 Mar 20;28:e76166. doi: 10.2196/76166 (PMC13006788; doi:10.2196/76166)
Supplement: Multimedia Appendix 1 [file jmir-v28-e76166-s001.docx]

**TUTORIAL – APPENDIX**

# Using an Adult-Designed Wearable for Pediatric Monitoring: A Practical Tutorial and Application in School-Aged Children with Obesity

## Data Import

Processing raw Fitbit intraday data requires manual inspection of file headers because these exports are not standardized across devices, firmware versions, or Fitbit/Google data-export platforms. As a result, MATLAB or Python import scripts must include conditional parsing logic or user-defined header mapping. Here we provide three headers from different studies as examples.

### Format of present study (research platform, Fitbit Charge 2/3, 2021)

{

"activities-heart": [...],

"activities-heart-intraday": {

"dataset": [

{ "time": "11:24:35", "value": 93 },

{ "time": "11:24:37", "value": 94 },

...

],

"datasetInterval": 1,

"datasetType": "second"

}

}

The dataset in the present tutorial is obtained from a research-level platform with an export function dedicated to researchers. In this format, intraday data are nested under “activities-heart-intraday”, PR value is stored under “value”, and timestamps are provided as “time” only, while the date is taken from the parent object. In the updated API format (~2024) the overall structure is maintained, but there are additional fields that make it necessary to double check the parsing.

### Format of Angelucci et al., JMIR, 2023 (consumer platform, Fitbit Inspire 2, 2021)

[

{

"dateTime": "06/10/21 12:36:36",

"value": { "bpm": 87, "confidence": 1 }

},

{

"dateTime": "06/10/21 12:36:41",

"value": { "bpm": 89, "confidence": 1 }

},

...

]

Differently from the data presented in this tutorial, the dataset in the 2023 paper [1] was downloaded from a consumer-grade platform rather than the research-grade platform provided by Fitbit. In this case, the same data could be downloaded for each user account with a more cumbersome procedure.

Here, there were no “activities-heart” or “intraday” fields, PR values were stored under “bpm” instead of “value”, and timestamps included both date and time in a single field (“dateTime”).

Because the formats differ in different elements, any generalized import script must first detect which structure is present and then map the fields accordingly. For this reason, users must manually inspect the header of each export file before applying the preprocessing pipeline.

## Supplementary Tables

**Table S1.** Quantitative wear time statistics per participant (mean of daily values ± standard deviation in the week of monitoring).

| **Participant** | **Total wear time (daily) [h]** | **Day wear time (daily) [h]** | **Night wear time (daily) [h]** | **>70% complete** |
| --- | --- | --- | --- | --- |
| G01 | 14.8 ± 3.0 | 13.1 ± 1.0 | 1.7 ± 2.9 | No |
| B02 | 8.9 ± 4.7 | 8.9 ± 4.6 | 0.0 ± 0.1 | No |
| G03 | 17.4 ± 5.7 | 11.1 ± 2.8 | 6.3 ± 3.1 | Yes |
| G04 | 12.6 ± 0.1 | 12.6 ± 0.1 | 0.0 ± 0.0 | No |
| B05 | 4.0 ± 4.9 | 4.0 ± 4.9 | 0.0 ± 0.0 | No |
| B06 | 23.1 ± 1.3 | 14.4 ± 1.3 | 8.7 ± 0.4 | Yes |
| B07 | 21.8 ± 3.7 | 14.1 ± 1.8 | 7.7 ± 2.6 | Yes |
| G08 | 1.2 ± 3.1 | 0.2 ± 0.4 | 1.0 ± 2.5 | No |
| G09 | 19.3 ± 6.2 | 12.2 ± 4.1 | 7.2 ± 2.9 | Yes |
| B10 | 8.6 ± 3.8 | 8.4 ± 3.8 | 0.2 ± 0.5 | No |
| B11 | 13.6 ± 3.9 | 11.3 ± 1.0 | 2.3 ± 3.3 | No |
| B12 | 11.0 ± 12.0 | 6.4 ± 7.9 | 4.6 ± 4.4 | No |
| B13 | 20.1 ± 4.8 | 12.4 ± 2.5 | 7.7 ± 2.6 | Yes |
| B14 | 17.8 ± 5.0 | 12.7 ± 1.8 | 5.1 ± 3.7 | Yes |
| B15 | 20.1 ± 4.1 | 12.5 ± 2.6 | 7.7 ± 2.5 | Yes |
| B16 | 16.6 ± 6.3 | 11.0 ± 4.1 | 5.7 ± 3.3 | No |

Participants with the code starting with ‘B’ are boys, while participants with the code starting with ‘G’ are girls. Statistics are reported using daily acquisitions, and standard deviation values are referred to the weekly distribution of the parameters.

**Table S2.** Multivariable linear regression models linking personal and wearable predictors with clinical outcomes. Unstandardized coefficients (β), standard errors (SE), and p-values are reported for all predictors included in each model. R² and adjusted R² refer to the full model. All p-values are unadjusted and should be interpreted as exploratory given the small sample size (n = 16, with some missing data).

| **Outcome** | **Predictor** | **β (Estimate)** | **SE** | **p-value** | **R^2^** | **Adjusted R^2^** | **n** |
| --- | --- | --- | --- | --- | --- | --- | --- |
| 6MWT | Intercept | -1500.6 | 1072.5 | 0.23 | 0.84 | 0.51 | 13 |
|  | Age | 40.53 | 40.21 | 0.37 |  |  |  |
|  | BMI | 2.32 | 19.73 | 0.91 |  |  |  |
|  | BMI Z-score | -109.96 | 176.99 | 0.57 |  |  |  |
|  | Mean steps | -0.01 | 0.01 | 0.29 |  |  |  |
|  | Max steps | -0.00 | 0.00 | 0.46 |  |  |  |
|  | RPR | 23.50 | 11.25 | 0.10 |  |  |  |
|  | RPR Z-score | -471.72 | 194.02 | 0.07 |  |  |  |
|  | PROS | -18.62 | 22.90 | 0.46 |  |  |  |
| 6MWT Z-score | Intercept | -44.49 | 13.01 | 0.027 | 0.93 | 0.79 | 13 |
|  | Age | 1.13 | 0.49 | 0.08 |  |  |  |
|  | BMI | -0.32 | 0.24 | 0.25 |  |  |  |
|  | BMI Z-score | 1.58 | 2.15 | 0.50 |  |  |  |
|  | Mean steps | -9.65 · 10^-5^ | 7.49 · 10^-5^ | 0.27 |  |  |  |
|  | Max steps | -4.63 · 10^-5^ | 5.24 · 10^-5^ | 0.43 |  |  |  |
|  | RPR | 0.46 | 0.14 | 0.03 |  |  |  |
|  | RPR Z-score | -9.11 | 2.35 | 0.02 |  |  |  |
|  | PROS | -0.14 | 0.28 | 0.63 |  |  |  |
| SAP | Intercept | 59.03 | 533.41 | 0.92 | 0.34 | -1.42 | 12 |
|  | Age | 2.08 | 32.06 | 0.95 |  |  |  |
|  | BMI | 1.40 | 19.96 | 0.95 |  |  |  |
|  | BMI Z-score | -6.13 | 192.19 | 0.98 |  |  |  |
|  | Mean steps | 0.00 | 0.00 | 0.59 |  |  |  |
|  | Max steps | 0.00 | 0.00 | 0.74 |  |  |  |
|  | RPR | -0.21 | 5.14 | 0.97 |  |  |  |
|  | RPR Z-score | 18.33 | 90.21 | 0.85 |  |  |  |
|  | PROS | -0.78 | 10.37 | 0.95 |  |  |  |
| SAP Z-score | Intercept | 238.79 | 1283.1 | 0.86 | 0.33 | -1.47 | 12 |
|  | Age | -4.00 | 77.11 | 0.96 |  |  |  |
|  | BMI | 6.44 | 48.01 | 0.90 |  |  |  |
|  | BMI Z-score | -36.87 | 462.31 | 0.94 |  |  |  |
|  | Mean steps | 0.00 | 0.01 | 0.78 |  |  |  |
|  | Max steps | 0.00 | 0.01 | 0.83 |  |  |  |
|  | RPR | -3.02 | 12.37 | 0.82 |  |  |  |
|  | RPR Z-score | 80.16 | 217.01 | 0.74 |  |  |  |
|  | PROS | -6.33 | 24.95 | 0.82 |  |  |  |

| **Outcome** | **Predictor** | **β (Estimate)** | **SE** | **p-value** | **R^2^** | **Adjusted R^2^** | **n** |
| --- | --- | --- | --- | --- | --- | --- | --- |
| DAP | Intercept | 246.49 | 229.46 | 0.36 | 0.71 | -0.08 | 12 |
|  | Age | -13.14 | 13.79 | 0.41 |  |  |  |
|  | BMI | 9.31 | 8.59 | 0.36 |  |  |  |
|  | BMI Z-score | -77.48 | 82.68 | 0.42 |  |  |  |
|  | Mean steps | 0.00 | 0.00 | 0.69 |  |  |  |
|  | Max steps | 0.00 | 0.00 | 0.85 |  |  |  |
|  | RPR | -1.74 | 2.21 | 0.49 |  |  |  |
|  | RPR Z-score | 43.18 | 38.81 | 0.35 |  |  |  |
|  | PROS | 1.34 | 4.46 | 0.78 |  |  |  |
| DAP Z-score | Intercept | 601.29 | 485.00 | 0.30 | 0.72 | -0.01 | 12 |
|  | Age | -31.53 | 29.15 | 0.36 |  |  |  |
|  | BMI | 19.15 | 18.15 | 0.37 |  |  |  |
|  | BMI Z-score | -145.53 | 174.75 | 0.47 |  |  |  |
|  | Mean steps | -0.00 | 0.00 | 0.95 |  |  |  |
|  | Max steps | -9.04 · 10^-5^ | 0.00 | 0.97 |  |  |  |
|  | RPR | -5.21 | 4.68 | 0.35 |  |  |  |
|  | RPR Z-score | 111.61 | 82.03 | 0.27 |  |  |  |
|  | PROS | 3.46 | 9.43 | 0.74 |  |  |  |

Models are underpowered (n = 12–13, 8 predictors) and should be interpreted as exploratory; negative adjusted R² in some models reflects small-n bias and overfitting.

**Table S3.** Predictors selected by LASSO with LOOCV for each outcome. For outcomes where no predictors were selected by LASSO, univariate fallback models are reported in Table 3 of the main text.

| **Outcome** | **LASSO-selected predictors (LOOCV)** |
| --- | --- |
| 6MWT | Age, BMI, Max steps, RPR Z-score |
| 6MWT Z-score | Age, BMI Z-score, Mean steps, Max steps, RPR, RPR Z-score, PROS |
| SAP | - |
| SAP percentile | Max steps |
| DAP | BMI |
| DAP percentile | - |

## References

1. Angelucci A, Greco M, Canali S, Marelli G, Avidano G, Goretti G, Cecconi M, Aliverti A. Fitbit Data to Assess Functional Capacity in Patients before Elective Surgery: Pilot Prospective Observational Study. J Med Internet Res 2023;25. doi: 10.2196/42815
